# Sex Disparities in Treatment Trajectories of Inflammatory Bowel Disease Are Associated With Diagnostic Delay

**DOI:** 10.1093/crocol/otaf040

**Published:** 2025-05-28

**Authors:** Lea Pueschel, Melanie Bathon, Ursula Seidler, Heiner Wedemeyer, Henrike Lenzen, Miriam Wiestler

**Affiliations:** Department of Gastroenterology, Hepatology, Infectious Diseases and Endocrinology, Hannover Medical School, Hannover, Germany; Department of Gastroenterology, Hepatology, Infectious Diseases and Endocrinology, Hannover Medical School, Hannover, Germany; Department of Gastroenterology, Hepatology, Infectious Diseases and Endocrinology, Hannover Medical School, Hannover, Germany; Department of Gastroenterology, Hepatology, Infectious Diseases and Endocrinology, Hannover Medical School, Hannover, Germany; Department of Gastroenterology, Hepatology, Infectious Diseases and Endocrinology, Hannover Medical School, Hannover, Germany; Department of Gastroenterology, Hepatology, Interventional Endoscopy and Diabetology, Academic Teaching Hospital Braunschweig, Braunschweig, Germany; Department of Gastroenterology, Hepatology, Infectious Diseases and Endocrinology, Hannover Medical School, Hannover, Germany; PRACTIS Clinician Scientist Program, Dean’s Office for Academic Career Development, Hannover Medical School, Hannover, Germany

**Keywords:** inflammatory bowel disease, diagnostic delay, treatment trajectories, sex differences

## Abstract

**Background:**

Diagnostic delay (DD) is a common finding in inflammatory bowel diseases (IBD). The reasons and effects of this delay are frequently underestimated, particularly in the context of sex. Our aims were to examine the impact of delayed diagnosis in IBD, with a particular focus on sex disparities.

**Methods:**

We performed a single-center, cross-sectional study at a tertiary referral center, including patients with IBD. The data was collected between August 2020 and June 2024.

**Results:**

A total of 247 individuals with IBD were included in this study, with 53% identifying as female and 51% having Crohn’s disease. Probability estimators revealed an effect of a DD on the cumulative advanced drug therapy probability in women (p_Log-Rank_ = 0.045). Further analysis of the interaction between therapeutic regimens and DD revealed significant differences between the sexes. Women with a longer latency in their diagnosis were more frequently treated with steroids only compared to men. Entity-specific DD was further identified as a risk factor for steroid-only treatment in women with IBD (OR: 2.6; 95% CI, 1.11-5.98; *P* = .028). Additionally, a notable disparity in quality of life was observed between women who exhibited DD and men, with the former demonstrating a significantly reduced quality of life.

**Conclusions:**

A delayed diagnosis has a significant impact on IBD treatment trajectories, with a notable sex-related effect observed especially in women. Therapeutic needs in female patients with IBD seem underestimated, particularly in instances where a DD is present.

## Introduction

Diagnostic delay (DD) is a well-recognized challenge in the management of inflammatory bowel diseases (IBD), encompassing Crohn’s disease (CD) and ulcerative colitis (UC). Despite the advancement of diagnostic techniques, the underlying reasons for delayed diagnosis remain to be fully elucidated.^1^ Studies have indicated that DDs are more protracted in CD compared to UC,^2^ a discrepancy that may be attributed to variations in disease manifestation and progression. Importantly, delayed diagnosis has significant implications, including worse disease outcomes, increased disease burden, and a heightened likelihood of requiring invasive interventions such as gastrointestinal (GI) surgery.^3–5^

Early and accurate diagnosis is therefore paramount in optimizing therapeutic strategies and improving long-term outcomes. Conversely, delays in diagnosis can lead to exacerbated disease progression,^3,4^ complicated management, and diminished quality of life for patients. The underlying causes of these delays are multifaceted, involving a complex interplay of patient-related, physician-related, and systemic factors. In the context of IBD, delays are most commonly observed between a patient’s initial presentation to a healthcare professional and the confirmation of a diagnosis. It is noteworthy that both sexes generally seek medical evaluation promptly following symptom onset,^6^ suggesting that delays are more attributable to factors within the diagnostic process itself.

Sex-based disparities in DDs have emerged as a pivotal yet under-explored research domain. Women with IBD have been documented to experience prolonged DDs in comparison to men, a discrepancy that has been observed in other medical contexts as well. These disparities may be attributed to biological factors, such as hormonal and genetic influences,^7–9^ or nonbiological factors, including disparities in access to healthcare, misdiagnosis, and the gender pain gap.^10,11^ Misdiagnosis with conditions such as irritable bowel syndrome (IBS) is particularly prevalent in women and may contribute to prolonged diagnostic timelines.

Understanding the relationship between DD, disease activity, and treatment trajectories is essential, particularly in light of sex-based variations. This study aims to investigate the association between DD and treatment trajectories in IBD, with a focus on sex-specific factors. By addressing this gap, we hope to inform strategies that minimize DDs, improve treatment outcomes, and reduce sex-based disparities in IBD care.

## Methods

### Participants and Setting

A total of 254 patients with IBD were screened with *n* = 247 subsequently enrolled at the IBD outpatient clinic of Hannover Medical School between August 2020 and June 2024. Prior to study inclusion, each patient was required to provide written informed consent. In order to be eligible for inclusion in the study, participants were required to meet the following criteria: a confirmed diagnosis of either UC or CD, disease duration of at least 3 months, and age between 18 and 75 years. Individuals with any disorders that preclude the assessment of the nature, scope, and potential consequences of the study were excluded. *N* = 7 individuals were excluded from the screening process as they had signed the ICF but did not provide further data or complete the screening questionnaire.

### Data Sources/Measurements

Data were collected via questionnaires and investigator-led interviews. Study participants completed a demographic survey, which included data on sex, body status (weight, height), and age. Patients were asked about their biological sex as well as their gender identity. Additionally, the treating physician independently verified the patient’s biological sex. The biological sex and gender identity of all study participants were identical for every individual. Furthermore, data on IBD-specific history, therapies, surgical history, and comorbidities were collected. The degree of disease activity was determined in investigator-led interviews by using either the German version of the Harvey-Bradshaw Index^12^ for patients with CD or the German version of the partial Mayo score^13^ for those with UC. To evaluate disease-related quality of life, patients were asked to fill out the 36-item German version of the inflammatory bowel disease questionnaire (IBDQ).^14,15^ Disease extent was determined using the Montreal Classification for patients with CD and the anatomic extent for patients with UC.^16^ Biomaterials, specifically blood and fecal samples, were collected and analyzed in a laboratory setting. The analysis included the measurement of fecal calprotectin (FC) (in mg/kg) and C-reactive protein (in mg/L).

### Sex and Gender

In the context of sex-related issues, the distinction between biological and nonbiological sex differences (eg, differences in treatment opportunities, the impact of healthcare systems, and differential access to care by sex)^17^ is essential. We therefore address putative sex-related differences in IBD treatment trajectories and management.

### Study Variables

#### Disease duration

Disease duration was defined as the time period between the initial diagnosis and the time of study inclusion.

#### Advanced drug therapy

The administration of advanced drug therapies (ADTs), including TNF-, interleukin (IL) 12/23 p40-, integrin antagonists, and Jak inhibitors, was documented for each patient.

#### Treatment trajectories

In order to identify potential deficiencies in the provision of care, a new variable, “treatment trajectories,” was created. This variable has 2 levels of detail, corresponding to the following categories: (1) patients who did receive treatment with ADTs at any point between the initial diagnosis and the time of study inclusion and (2) patients who received only steroids throughout the course of their disease. For a more detailed analysis, the variable “therapy course” was created with 4 levels of detail: (1) patients who did not receive any treatment with ADTs and/or corticosteroids; (2) patients who received only ADTs; (3) patients who received only corticosteroids; and (4) patients who were treated with both ADTs and corticosteroids throughout the course of their disease.

#### Diagnostic delay

To ascertain the length of DD, the time between the first onset of symptoms and the diagnosis of IBD (diagnostic latency) was evaluated using median delay values specific to each entity, with a cutoff value of 12 months for CD and 6 months for UC based on the findings of a systematic review by Cross et al. which encompasses 31 manuscripts.^2^

#### Gastrointestinal surgery

The status of gastrointestinal (GI) surgery was documented and subsequently employed as a binary variable (GI surgery = yes or no) in instances pertaining solely to surgeries associated with IBD.

### Statistical Analyses

The statistical analysis was conducted using the SPSS Statistics software, version 29.0.1.0 (SPSS, IBM, Armonk, NY), and GraphPad PRISM, version 10.4.0 (GraphPad Software, Boston, Massachusetts, USA). Categorical baseline characteristic variables are described in terms of total and percentage values. Group comparison was conducted using Student’s *t*-test. Where possible effect size is reported (either as (g) or (f)) in addition to the level of significance (*P*). For Fisher’s exact test the odds ratio (OR) is also reported. Unless otherwise indicated, all statistical tests were 2-sided. In order to analyze the time to event of treatment trajectories over disease duration, we employed the Log-Rank (Mantel–Cox) test as a comparison parameter for DD. Subsequently, the data were stratified according to sex and analyzed using multivariate logistic regression (backwards stepwise: Wald) to evaluate the probability of disease-related stationary factors in relation to steroids-only treatment. Odds ratio, 95% confidence interval (CI), and the level of significance (*P*) are reported. Goodness of fit for the fully adjusted logistic regression model for IBD women with the outcome steroids only was assessed via Omnibus Tests of Model Coefficients (*P* = .001), *R*^2^ (Nagelkerkes: 0.166; Cox & Snell: 0.115), and the Hosmer–Lemeshow test (*P* = .308). The model performance was assessed via the classification table, which showed an overall percentage of 72.7%. Goodness of fit for the fully adjusted logistic regression model for IBD men with the outcome steroids only as well as the corresponding results can be found in the supplementary data (Table S1).

#### Confounders

In order to control for potential confounding variables, the regression models were adjusted for established confounding factors, including disease entity, surgical status, extraintestinal manifestation, and age. The incorporation of variable data, including laboratory values such as FC, was deliberately eschewed in the model’s formulation. Instead, the regression model was exclusively comprised of static data elements, such as disease entities.

#### Bias

In a questionnaire-based enquiry all study subjects were asked to provide dates of onset of first symptoms and initial diagnosis. It is possible that the results may be affected by recall bias. In cases where the date of symptom onset and the date of IBD diagnosis were documented in the medical record, these dates have been confirmed. However, verification was not possible for all cases.

To exclude the SARS-CoV-2 pandemic as an influencing bias factor on delayed diagnosis, we evaluated the data accordingly. The diagnosis date of all participants was recoded as a binary variable, with the occurrence of Germany’s initial lockdown period used as the cutoff point (diagnosis prior to March 2020; diagnosis post-March 2020). A numerical trend emerged: 28 (*n* = 13 women) of the 247 patients received their diagnosis post-March 2020, with the mean time from symptom onset to diagnosis being 18 months. For patients who received their diagnosis before the pandemic though, the mean time was 16 months. However, these differences did not reach statistical significance. It was further noted that no patient with the potential for childbearing experienced a pregnancy during the course of the study.

## Results

### Study Population

A total of 247 patients were enrolled in this study (*n* = 130 women) out of 254 patients who were screened. The baseline characteristics were largely well balanced between the groups, with differences observed in the absolute distribution of men and women between the entities with a shift towards female patients with CD (59.2% vs 41.9%) and male patients with UC (58.1% vs 40.8%). Median age (men: 38 [IQR:28-51] years vs women: 36 [IQR: 28-47] years) and disease duration (men: 10.76 [IQR: 4.69-19.64] years vs women: 11.02 [IQR: 5.98-18.02] years) were well balanced between the groups. In terms of diagnostic latency (men: 5 [IQR: 3-14] months vs women: 6 [IQR: 4-16] months) as well as DD (men: 45.3% vs 46.2%) numerical trends towards longer delays have been found in female patients with IBD. No further significant differences were observed between the groups with regard to disease-specific aspects, including disease activity, disease location, disease behavior, and therapeutic regimens (Table 1).

**Table 1.** Baseline characteristics stratified by sex.

		Men	Women	
		(*n* = 117)	(*n* = 130)	p_Sex_
Entity [*n* (%)]	Crohn’s Disease	49 (41.9%)	77 (59.2%)	**0.008**
	Ulcerative Colitis	68 (58.1%)	53 (40.8%)	**0.008**
Disease activity [*n* (%)]	Remission	45 (38.8%)	60 (46.9%)	0.204
	Mild Disease	41 (35.3%)	31 (24.2%)	0.057
	Moderate Disease	25 (21.6%)	30 (23.4%)	0.726
	Severe Disease	5 (4.3%)	7 (5.5%)	0.674
Location of Crohn’s [*n* (%)]	L1: ileal	16 (32.7%)	34 (43.6%)	0.219
	L2: colonic	10 (20.4%)	11 (14.1%)	0.352
	L3: ileocolonic	22 (44.9%)	32 (41%)	0.667
	L4: isolated upper disease	1 (2%)	1 (1.3%)	0.741
Crohn’s behavior [*n* (%)]	B1: nonstricturing, nonpenetrating	13 (26.5%)	27 (34.6%)	0.342
	B2: stricturing	18 (36.7%)	27 (34.6%)	0.810
	B3: penetrating	18 (36.7%)	24 (30.8%)	0.484
UC Montreal classification [*n* (%)]	Proctitis	2 (2.9%)	2 (3.8%)	0.803
	left-sided colitis	23 (33.8%)	24 (45.3%)	0.201
	pancolitis	43 (63.2%)	27 (50.9%)	0.174
Disease duration in years [median (IQR)] (years)		10.76 (4.69-19.64)	11.02 (5.98-18.02)	0.952
Diagnostic latency [median (IQR)] (months)		5 (3-14)	6 (4-16)	0.490
Diagnostic delay (outside benchmark range) [*n* (%)]		53 (45.3%)	60 (46.2%)	0.899
Extraintestinal Manifestation [*n* (%)]		53 (45.3%)	75 (57.7%)	0.057
IBDQ score [median (IQR)]		153 [120-173]	146 [105-170]	**0.019**
Advanced therapies (current or former) [*n* (%)]		81 (69.2%)	95 (73.1%)	0.574
Steroids (current or former) [*n* (%)]		105 (89.7%)	113 (86.9%)	0.556
BMI [median (IQR)] (kg/m^2^)		24 (21.22-27.50)	23.12 (21-27)	0.802
Age [median (IQR)] (years)		38 (28-51)	36 (28-47)	0.330
Smoking status (current or former) [*n* (%)]		33 (28.2%)	43 (33.3%)	0.410
Calprotectin [median (IQR)] (mg/kg)		201 (42.9-1410)	165 (58.4-790)	0.852
C-reactive protein [median (IQR)] (mg/L)		1.9 (0.7-5.9)	2.4 (0.9-6.45)	0.486

Values as totals and percentages [*n* (%)] or median and interquartile range [Mdn. (IQR)]. Bold font indicates statistical significance.

Abbreviations: BMI, body mass index; IBDQ, inflammatory bowel disease questionnaire; UC, ulcerative colitis.

### Sex-Specific Impact of DD on the Treatment Trajectories of IBD

**Figure 1.**
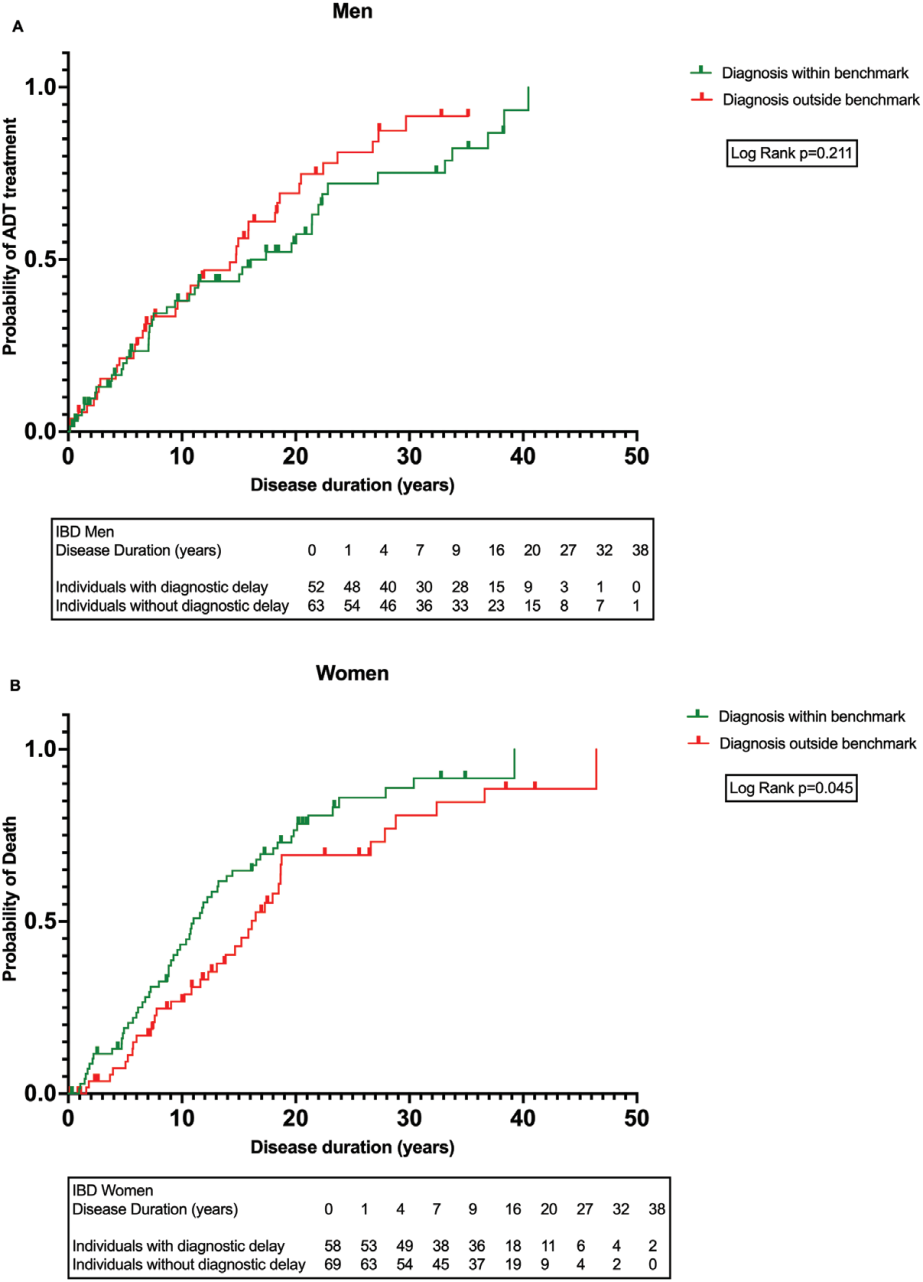
(A) and (B) Kaplan–Meier analysis (one minus survival function) demonstrating ADT treatment probability over disease duration grouped by DD. (A) Men: curves for DD; (B) Women: curves for DD. Abbreviation: ADT, advanced drug therapy.

To assess the disparate impacts of DD, Kaplan–Meier estimators were employed to contrast the likelihood of receiving ADT over the disease duration between 2 cohorts: those with diagnoses within the benchmark range (no DD) and those with diagnoses outside the benchmark range (DD). This analysis was conducted with data stratified by sex. Women with DD exhibited significantly lower ADT probabilities in comparison to those diagnosed within the benchmark range (p_Log-Rank_ = 0.045). This phenomenon was not observed in men (p_Log-Rank_ = 0.211) (Figure 1A and B).

### Sex-Related Differences Between IBD Treatment Trajectories and Diagnostic Latency

**Figure 2.**
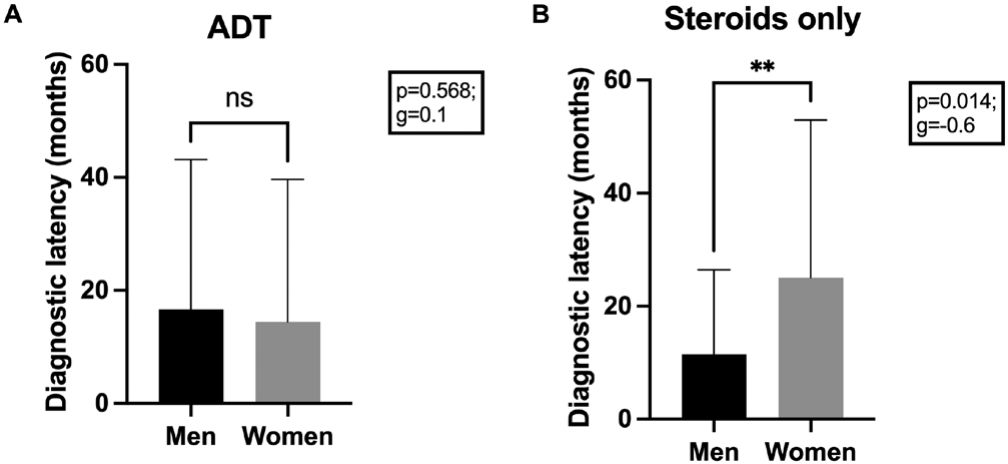
(A) and (B) IBD treatment trajectories and diagnostic latency. Results of Student’s *t*-test between (A) men/women groups for diagnostic latency (months) and ADT (*P* = .568; *g* = 0.1), and (B) men/women groups for diagnostic latency (months) and steroids only (*P* = 0.014; *g* = −0.6). Abbreviation: ADT, advanced drug therapy.

In order to examine the potential for sex-related disparities in the treatment trajectories of individuals diagnosed with IBD in the context of diagnostic latency, Student’s *t*-test was used. Group comparison revealed notable sex-related discrepancies in the treatment trajectories over diagnostic latency (in months). The differences between men and women were most pronounced for those who were ADT-naïve, with the mean time from symptom onset to diagnosis was reported as 25.0 months for women who had only received steroids and 11.5 months for men who had only received steroids (*P* = .0014; *g* = −0.6). There was no significant difference in diagnostic latency between the sexes who received ADTs (women: 14.4 months; men: 16.6 months; *P* = .568; *g* = −0.1) (Figure 2A and B).

A subsequent investigation was conducted to identify entity-specific trends in sex-related differences over the mean time from symptom onset to diagnosis for the overall treatment patterns, corticosteroid treatment, and the number of different ADTs. However, the analysis revealed that most of the resulting subgroups were too small to ensure the validity of the findings (Figure S1A–F). Nevertheless, the trend indicated that men with CD and UC as well as women with UC with the longest mean time from symptom onset to diagnosis (CD men: 48 months; UC men: 46 months; UC women: 25.4 months) were treated with ADT only while women with CD with the longest mean time from symptom onset to diagnosis (38 months) were treated with corticosteroids only. Additionally, women with CD who reported the second longest timeframe of 32.3 months received neither treatment with ADTs nor corticosteroids (Figure S1A–F).

### Influence of Stationary Disease-Specific Factors on the Treatment Trajectories in Men and Women With IBD

To determine the impact of stationary IBD-related factors on sex-specific treatment trajectories an adjusted logistic regression analysis was conducted. The influence of steady disease-specific factors on treatment trajectories showed no statistically significant risk factors for men with IBD (Table S1). The same analysis showed that DD increased the risk of steroid-only treatment for women (OR: 2.6; 95% CI, 1.11-5.98; *P* = .028). The likelihood for steroid-only treatment was also significantly increased in women without GI surgery (OR: 2.9; 95% CI, 1.05-8.14; *P* = .040). Age was also shown to be a statistically significant risk factor (OR: 1.0; 95% CI, 1.00-1.07; *P* = .030) (Table 2).

**Table 2.** The impact of disease-specific influencing factors on the outcome of steroid-only treatment in women with IBD.

		IBD Women—Outcome: Steroid treatment only		
		*n*	Fully adjusted OR [95% CI]	p
GI surgery	Yes (1)	38	2.9 [1.05-8.14]	**0.040**
	No	90		
DD	Yes	58	2.6 [1.11-5.98]	**0.028**
	No (1)	70		
Age		128	1.0 [1.00-1.07]	**0.030**

Results of logistic regression analysis (adjusted(multivariate)) are reported as the OR, 95% CI, and level of significance (*P*). Bold font indicates statistical significance.

Adjustment factors for the fully adjusted model: disease entity, disease duration (years), extraintestinal manifestations; age; disease entity-specific DD, gastrointestinal surgery.

Abbreviations: IBD, inflammatory bowel disease; GI, gastrointestinal; DD, diagnostic delay.

### Disease-Related Quality of Life in Relation to DD Between the Sexes

**Figure 3.**
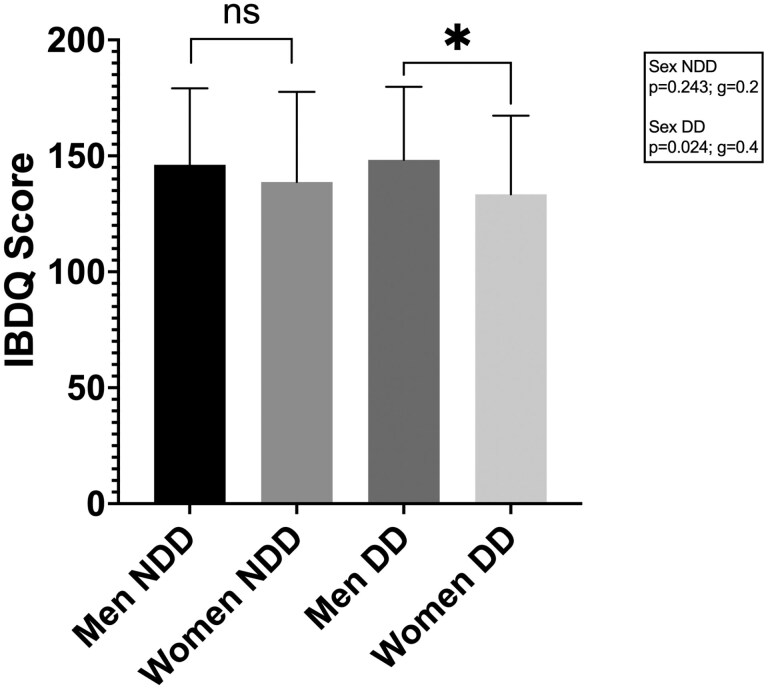
Disease-related quality of life in relation to DD between the sexes. Results of Student’s *t*-test between men/women groups without a diagnostic delay (NDD: *P* = 0.243; *g* = 0.2), and men/women with a diagnostic delay (DD: *P* = .024; *g* = 0.4). Abbreviations: DD, diagnostic delay; IBDQ, inflammatory bowel disease questionnaire; NDD, no diagnostic delay.

In order to further investigate the patients´ relevance of a DD on the level of disease-related quality of life a distinct group comparison between the sexes with and without DD was employed. Results of Student’s *t*-test between the sexes without a DD showed no difference in IBDQ mean (NDD: *P* = .243; *g* = 0.2). However, disease-related quality of life differed significantly between men and women with a DD (*P* = .024; *g* = 0.4) (Figure 3).

## Discussion

In the context of personalized medicine in IBD, a more profound comprehension of the sex-related elements of diagnosis, disease progression, and therapeutic management is imperative. Accordingly, the objective of this study was to conduct a comprehensive analysis of the impact of sex-related disparities on the association between DDs in IBD and further therapeutic strategies. Herewith, we wish to sensitize on this explicit topic of sex disparities with the aim of optimizing the care of all individuals with IBD. The findings of this study represent a significant expansion of the existing knowledge base on sex-related differences in patients with IBD with regard to the clinical impact of a DD. Here, we illustrate that women with a DD are more likely to be treated with steroids only compared to men.

While the sex distribution within our collective exhibited discrepancies, our analysis did not yield statistically significant differences but numerical trends in the diagnostic latency between the sexes. A recent analysis of DD in a German IBD cohort yielded results that were in contrast to other studies^6,18^ as they did not identify female sex as a risk factor for DD, which corresponds to the results observed in our study.^19^ But interestingly we identified entity-specific DD as a risk factor against ADT use in IBD women even though entity-specific DD did not discriminate between the sexes. It can thus be assumed that other underlying factors are relevant, including a consistent and systemic sex bias in the diagnostic process and further disease course, which is to the detriment of female patients. A possible reason for this could be that female patients are more likely to be initially misdiagnosed than male patients^6^ as well as having their symptoms dismissed due to preexisting diagnoses of IBS or depression. Depression is more common in women with IBD than in men,^20^ as is a premorbid diagnosis of IBS,^21^ with both however being considered a risk factor against a timely diagnosis.^22^ Misdiagnosis as well as dismissal of symptoms may well indicate the presence of a systematic diagnostic bias with regard to digestive symptoms and sex, whereby symptoms are more frequently attributed to functional rather than organic disorders in women.^6^

Our subsequent findings indicated that women with the longest DD are currently treated exclusively with steroids. This is in contrast to the general IBD treatment recommendations, which do not include corticosteroids as a maintenance therapy option.^23^ In line with these considerations, our analysis demonstrated on the other hand that men with higher DDs were more likely to receive ADT rather than steroid therapy which is consistent with the general recommendations for the treatment of IBD. Bearing in mind the sex disparities in IBD entity, phenotype and progression, male sex is indeed associated with an increased risk of early disease onset, upper GI involvement, penetrating behavior, perianal disease, complications,^24^ primary sclerosing cholangitis, colectomy, developing colorectal cancer,^25^ as well as postoperative disease recurrence.^26^ In consideration of the male-predominant risks, our findings indicate that the relevant treatment courses for male patients are implemented in a manner that aligns with the prevailing recommendations in the field. However, to control for possible confounders we analyzed disease-specific factors for all individuals with a DD, revealing that in our cohort disease phenotype in cases of DD were mostly balanced between the sexes. Our data demonstrated that CD men did not present with more severe or complicated cases. The majority of CD men and women were classified as Montreal B2 (37.5%; CD women: 45.5%) as well as having no concomitant perianal disease (87.5%; CD women: 87.9%). For patients diagnosed with colitis, the majority exhibited an extensive disease phenotype (pancolitis: 72.4% of male patients; 55.6% of female patients). However, this discrepancy in distribution was not statistically significant (*P* = .999).

Meanwhile, we found that the likelihood of women receiving ADT treatment decreases with increasing age, thus confirming the results of a recent Canadian cohort study.^27^ While the increased occurrence of side effects during ADT treatment in women and the presumably resulting lack of treatment adherence have been documented,^28,29^ this cannot explain the sex-specific differences in the ADT-naive collective. The fully adjusted analysis revealed additional effects, particularly that the likelihood for steroid-only treatment was significantly increased in women with a DD as well in women without gastrointestinal surgery. While women without a DD who had undergone GI surgery had a 1.33 higher probability of ADT treatment than women without GI surgery this effect was nearly quadrupled for women with a DD. Postsurgical therapeutic management of IBD is of upmost importance; however, there is no consent regarding the specific treatment.^30,31^ It is imperative to consider the impact of symptom negotiation in female patients, given the systemic tendency to attribute symptoms to functional rather than organic disorders. Thus, the absence of surgical intervention could potentially result in a reduction in disease progression awareness among physicians. Sex differences in IBD prescription of immunosuppressive therapies have been described before^32^ and are in consent with our findings. Potential explanations for these findings include a higher risk of developing more severe disease in males, lower compliance with corticosteroids and/or aminosalicylates in males, and avoidance of immunosuppressive agents in females of childbearing age. Our findings further demonstrated a significant reduction of disease-related quality of life especially in females with a DD. We see within our cohort clear numerical trends of an overall decreased disease-related quality of life in female individuals with IBD compared to males. This in in line with general findings demonstrating higher psychosocial burden of female individuals with IBD with a significant decrease in their perceived quality of life.^33^ Our findings reveal that a DD further aggravates this decrease in female patients’ quality of life thereby demonstrating the global relevance of our findings on a qualitative patients level.

In light of the aforementioned unfavorable treatment trajectories in IBD women with a DD, we hypothesize another level of these biases, which indicate an upstream, systematic, sex-biased underestimation of IBD symptoms especially in women. A first measure to assist in combating the observed sex bias in the medical assessment of IBD symptoms may well be the increased utilization of objective diagnostics and biomarkers, such as FC. While it has been shown that appropriate FC testing decreases the time to diagnosis^18^ the available data on sex-biases is nevertheless limited, and it must be noted that calprotectin can also be elevated in other differential diagnoses. Therefore, further objective diagnostic parameters to distinguish between IBS and IBD would be required, such as the 8-item questionnaire (CalproQuest) which can help to identify potential patients with IBD.^34^ Furthermore, since a preexisting diagnosis of IBS has been identified as a risk for an IBD DD^22^ tools to discriminate between IBD and differential diagnosis such as the “Red Flags Index for Suspected CD”^35^ could be beneficial. In the absence of additional validated objective diagnostic tools, however, it is important to be aware of the growing evidence of sex disparities in IBD, especially since their implications remain underestimated as of today.

As this was a single-center, cross-sectional study, it is not possible to discount the possibility of inclusion biases. Although our data illustrate the necessity for analyses to be conducted not only on an entity-specific basis but also on a sex-specific basis, the resulting subgroups were insufficiently populated to represent all possible populations, particularly given the limitations of a small sample size (eg, UC classification E1: proctitis). However, it must be stressed that in particular disease phenotype and severity are possible confounders and that the analysis is lacking in this instance. Moreover, the available data did not allow to differentiate between reasons for DDs such as a delay in help-seeking of the patient after symptom onset, or a physician/healthcare-related delay in the diagnostic process. One further potential confounding factor is physician sex; however, the sex of the treating physician for the course of disease and treatment of the individual patients is not documented. This limitation is primarily attributable to the nature of the study center, which functions as a referral center, providing treatment to patients in multiple different practice settings. In addition, a notable constraint pertains to the paucity of data concerning pregnancy. It is plausible that pregnancy could serve as a confounding factor, potentially influencing the observed delay in the commencement of ADT among the childbearing demographic. Finally, despite the statistical significance of our findings, the underlying core of these disparities cannot ultimately be answered, as qualitative data on, eg, symptom characteristics and corresponding actions taken were not explicitly analyzed. As this provides an opportunity for further evaluations of these specific effects with the inclusion of a greater number of patients, we have initiated a nationwide multicenter study in order to evaluate this question comprehensively and decisively.

In conclusion, this study concentrated on the sex-related interplay between DD and further disease-related factors. Our findings demonstrated a significant influence of sex on the relationship between DD and treatment trajectories. These phenomena were particularly evident in IBD women, indicating the potential for a sex-related bias. In order to achieve these objectives and within the context of precision medicine, it is imperative to emphasize the necessity for a more personalized approach to healthcare that considers the sex-specific differences in the presentation and management of IBD. This approach has the potential to result in improved outcomes for both men and women with IBD.

## Supplementary Material

otaf040_suppl_Supplementary_Table_S1

otaf040_suppl_Supplementary_Figure_S1

## Data Availability

Data, analytic methods, and study materials will be made available on specific request.
