# Supplementary material for: Sex Disparities in Treatment Trajectories of Inflammatory Bowel Disease Are Associated With Diagnostic Delay
Source: Crohns Colitis 360. 2025 May 28;7(3):otaf040. doi: 10.1093/crocol/otaf040 (PMC12260162; doi:10.1093/crocol/otaf040)
Supplement: otaf040_suppl_Supplementary_Table_S1 [file otaf040_suppl_supplementary_table_s1.docx]

| Supplementary Table 1 |  | **IBD Men - Outcome: Steroid Treatment Only** | | |
| --- | --- | --- | --- | --- |
|  |  |  |  |  |
|  |  | **n** | **Fully Adjusted OR [95% CI]** | **p** |
| Entity | CD | 49 | 0.5 [0.20 - 1.07] | 0.073 |
|  | UC (1) | 68 |  |  |
| EIM | none (1) | 64 | 0.5 [0.20 - 1.04] | 0.062 |
|  | EIM | 53 |  |  |
|  | | | | |

**Supplementary Table 1:** Results of logistic regression analysis (adjusted(multivariate) are reported as the odds ratio (OR), 95% confidence interval (CI), and level of significance (p).

Adjustment factors for the fully adjusted model: disease entity, disease duration (years), extraintestinal manifestations; age; disease entity-specific diagnostic delay, gastrointestinal surgery.

IBD – inflammatory bowel disease; GI – gastrointestinal; DD – diagnostic delay.

Omnibus Tests of Model Coefficients (p=0.040), R2 (Nagelkerkes: 0.075; Cox & Snell: 0.053), and the Hosmer–Lemeshow test (p = 0.999). Model performance: 69.2%.

**Supplementary Figure 1:** Sex-related differences over the mean time from symptom onset to diagnosis for the overall treatment patterns, corticosteroid treatment, and the number of different ADTs.

ADT - advanced drug therapy.
