# Supplementary figures and images for: Sex Disparities in Treatment Trajectories of Inflammatory Bowel Disease Are Associated With Diagnostic Delay
Source: Crohns Colitis 360. 2025 May 28;7(3):otaf040. doi: 10.1093/crocol/otaf040 (PMC12260162; doi:10.1093/crocol/otaf040)

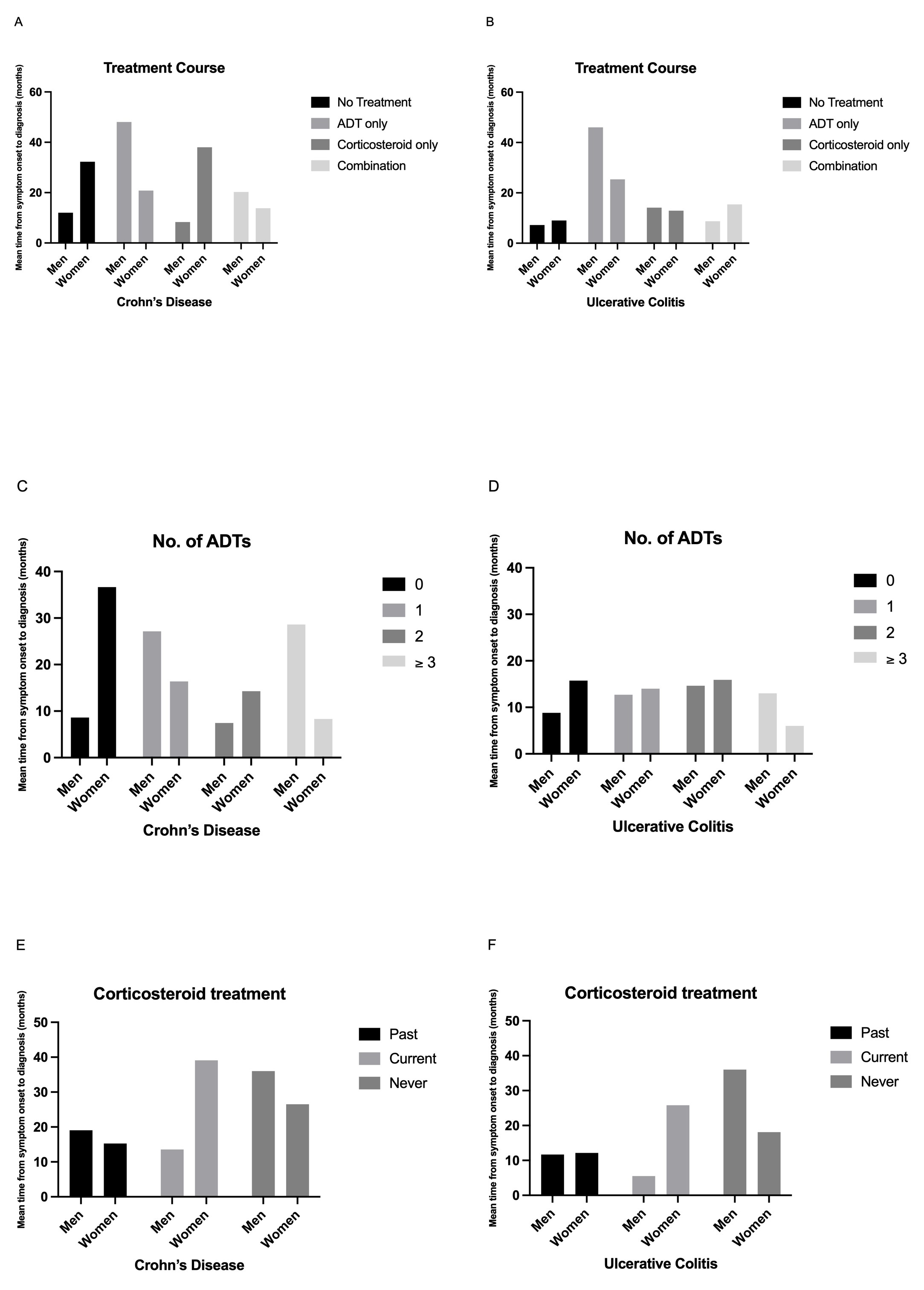

Supplement: otaf040_suppl_Supplementary_Figure_S1 [file otaf040_suppl_supplementary_figure_s1.jpeg]
